# Diaphragm stimulation in critically ill patients: what do we know, and what do we still need to learn?

**DOI:** 10.31744/einstein_journal/2026CE2373

**Published:** 2026-05-21

**Authors:** Julia Machado da Costa Pennone, Carolina Pereira Batista, Giulia Alves Salustiano Polloni, Marina Brunelli Ferreira Bueno, Angélica Cristiane da Cruz Britto, Ricardo Kenji Nawa, Caroline Gomes Mól

**Affiliations:** 1 Hospital Israelita Albert Einstein Faculdade Israelita de Ciências da Saúde Albert Einstein São Paulo SP Brazil Faculdade Israelita de Ciências da Saúde Albert Einstein, Hospital Israelita Albert Einstein, São Paulo, SP, Brazil.; 2 Hospital Israelita Albert Einstein São Paulo SP Brazil Hospital Israelita Albert Einstein, São Paulo, SP, Brazil.

Dear Editor,

Diaphragm weakness is a common complication in critically ill patients and can develop within days after the initiation of invasive mechanical ventilation.^(1,2)^ It is characterized by a rapid loss of diaphragm contractile capacity, which compromises the patient's ability to breathe independently.^(1,2)^ Diaphragm weakness is strongly associated with worse clinical outcomes, including prolonged weaning from mechanical ventilation (MV), longer intensive care unit (ICU) and hospital length of stay, and higher mortality.^(1-4)^ Because of its high prevalence and major clinical impact, substantial efforts have focused on identifying strategies and interventions to prevent or reverse diaphragm weakness and dysfunction, such as inspiratory muscle training, early mobilization, and diaphragm electrical stimulation.^(5-8)^

In this context, neuromuscular stimulation has emerged as a promising strategy to preserve or restore diaphragmatic function in critically ill patients, either through direct stimulation of the diaphragm muscle or by stimulating the phrenic nerve, which innervates the diaphragm.^(6,9)^ The rationale behind these techniques is to preserve diaphragm activity during MV, thereby limiting disuse atrophy and contractile dysfunction. Transcutaneous stimulation is commonly described as the application of electrical stimuli through surface electrodes placed on the thoracoabdominal region to directly recruit the diaphragm at the zone of apposition, where it lies closer to the body surface.^(6,10,11)^ Another non-invasive approach involves cervical stimulation using surface or pen electrodes along the course of the phrenic nerve to indirectly activate the diaphragm.^(9)^ Invasive approaches, including intramuscular diaphragmatic pacing and transvenous phrenic nerve stimulation, have also been investigated and may provide more targeted and sustained stimulation; however, their implementation in clinical practice is associated with greater procedural complexity.^(6,12)^

To date, the available evidence indicates that diaphragm stimulation is feasible and generally safe, with preliminary studies suggesting potential benefits such as shorter duration of MV, reduced ICU length of stay, and improved success of ventilator weaning in patients requiring prolonged MV.^(6,9-12)^ However, most of these data come from small and methodologically heterogeneous studies, which limits the strength and generalizability of these findings. As a result, although diaphragm stimulation appears to be a promising strategy for preventing and treating diaphragm dysfunction in critically ill patients, its role in routine clinical practice and its true impact on patient-centered outcomes remains insufficiently established. Despite the growing body of evidence supporting diaphragmatic stimulation in critically ill patients, important knowledge gaps remain. These include identifying which patient populations are most likely to benefit and comparing the effectiveness of direct diaphragmatic versus phrenic nerve stimulation, defining optimal stimulation parameters, and establishing how best to integrate these interventions into standard respiratory and rehabilitation protocols.

The impact of diaphragmatic stimulation on clinically meaningful short- and long-term outcomes remains poorly defined. Well-designed, prospective clinical trials are therefore needed to clarify the therapeutic value of diaphragmatic stimulation and to support the development of evidence-based guidelines for its implementation in ICUs worldwide.

## Data Availability

The underlying content is contained within the manuscript.
